# Crystal structure of MbnF: an NADPH-dependent flavin monooxygenase from *Methylocystis* strain SB2

**DOI:** 10.1107/S2053230X23003035

**Published:** 2023-05-05

**Authors:** Andrew Stewart, Philip Dershwitz, Charles Stewart, Michael R. Sawaya, Todd O. Yeates, Jeremy D. Semrau, Hans Zischka, Alan A. DiSpirito, Thomas A. Bobik

**Affiliations:** a Roy J. Carver Department of Biochemistry, Biophysics and Molecular Biology, Iowa State University, Ames, IA 50011-3260, USA; b Macromolecular X-ray Crystallography Facility, Office of Biotechnology, Iowa State University, Ames, IA 50011-3260, USA; c UCLA–DOE Institute for Genomics and Proteomics, University of California Los Angeles, Los Angeles, CA 90095-1570, USA; d Department of Civil and Environmental Engineering, University of Michigan, Ann Arbor, MI 48109-2125, USA; e Institute of Molecular Toxicology and Pharmacology, Helmholtz Center Munich, German Research Center for Environmental Health, Ingolstaedter Landstrasse 1, 85764 Neuherberg, Germany; f School of Medicine, Institute of Toxicology and Environmental Hygiene, Technical University Munich, Biedersteiner Strasse 29, 80802 Munich, Germany; University of Leipzig, Germany

**Keywords:** methanobactins, MbnF, flavin monooxygenases, *Methylocystis* sp. strain SB2, Wilson’s disease

## Abstract

Methanobactins (MBs) are ribosomally produced and post-translationally modified peptides (RiPPs) that are used by methanotrophs for copper acquisition. The signature post-translational modification of MBs is the formation of two heterocyclic groups, either an oxazolone, pyrazinedione or imidazolone group, with an associated thioamide from an *X*-Cys dipeptide. The precursor peptide (MbnA) for MB formation is found in a gene cluster of MB-associated genes. The exact biosynthetic pathway of MB formation is not yet fully understood, and there are still uncharacterized proteins in some MB gene clusters, particularly those that produce pyrazinedione or imidazolone rings. One such protein is MbnF, which is proposed to be a flavin monooxygenase (FMO) based on homology. To help to elucidate its possible function, MbnF from *Methylocystis* sp. strain SB2 was recombinantly produced in *Escherichia coli* and its X-ray crystal structure was resolved to 2.6 Å resolution. Based on its structural features, MbnF appears to be a type A FMO, most of which catalyze hydroxylation reactions. Preliminary functional characterization shows that MbnF preferentially oxidizes NADPH over NADH, supporting NAD(P)H-mediated flavin reduction, which is the initial step in the reaction cycle of several type A FMO enzymes. It is also shown that MbnF binds the precursor peptide for MB, with subsequent loss of the leader peptide sequence as well as the last three C-terminal amino acids, suggesting that MbnF might be needed for this process to occur. Finally, molecular-dynamics simulations revealed a channel in MbnF that is capable of accommodating the core MbnA fragment minus the three C-terminal amino acids.

## Abstract 2

Methanobactins are post-translationally modified copper-binding peptides that have a number of potential environmental and biomedical applications. This report presents the crystal structure and preliminary biochemical characterization of the putative methanobactin biosynthesis protein MbnF.

## 1. Introduction

It has recently been shown that akin to the siderophores that are used for iron uptake (Neilands, 1995 ▸), some microbes produce chalkophores or copper-binding compounds for copper uptake (DiSpirito *et al.*, 2016 ▸; Semrau *et al.*, 2020 ▸). The best-characterized chalkophores are methanobactins (MBs) from methane-oxidizing bacteria (methanotrophs). MBs are small (less than 1300 Da) post-translationally modified peptides with two heterocyclic groups, either an imidazole, oxazolone or pyrazinedione group, each of which is associated with a thioamide formed from an *X*C dipeptide. These heterocyclic groups, with the associated thioamide group, are responsible for copper binding (El Ghazouani *et al.*, 2011 ▸, 2012 ▸; Kim *et al.*, 2004 ▸).

**Figure 1:**
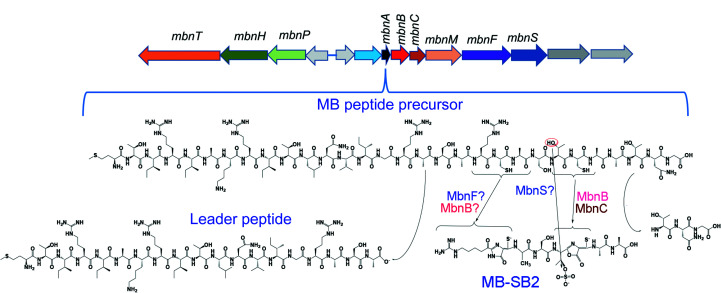
Top: structural genes for the synthesis and use of MB from *Methylocystis* sp. strain SB2: *mbnA* (encodes the MB-SB2 precursor peptide), *mbnB* (encodes a DUF683 di-iron enzyme), *mbnC* (an unannotated gene involved in oxazolone-group formation), *mbnT* (encodes a TonB transporter), *mbnS* (encodes a putative sulfotransferase), *mbnF* (encodes a FAD-dependent oxidoreductase), *mbnH* (encodes a di-heme cytochrome *c* peroxidase), *mbnP* (encodes the partner of *mbnH*) and *mbnM* (encodes a multidrug antimicrobial extrusion protein). Bottom: the MB-SB2 peptide precursor which is cleaved and processed to form MB-SB2. Also shown are known or proposed roles of Mbn proteins in the biosynthesis of MB-SB2 from the MB precursor peptide.

There is a great deal of interest in determining how mature MBs are formed from a polypeptide precursor as MBs have significant medical and environmental applications (Fig. 1 ▸; Baral *et al.*, 2014 ▸; Johnson, 2006 ▸; Lu *et al.*, 2017 ▸; Semau *et al.*, 2020 ▸; Vorobev *et al.*, 2013 ▸; Chang *et al.*, 2018 ▸, 2022 ▸; Einer *et al.*, 2019 ▸; Kang-Yun *et al.*, 2022 ▸; Lichtmannegger *et al.*, 2016 ▸; Zischka *et al.*, 2011 ▸). The proteins involved in the formation of the C-terminal oxazolone group and the associated thioamide have been identified and characterized via selective knockouts of genes in the MB gene cluster (Gu *et al.*, 2017 ▸; Dershwitz *et al.*, 2022 ▸) as well as heterologous expression (Kenney *et al.*, 2018 ▸). Little is known, however, about how the tautomeric imidazole/pyrazinedione N-terminal group is formed or how the leader sequence is removed to form mature MB. Interestingly, MbnF is present in all methanobactin-biosynthesis operons known to produce MB with an N-terminal imidazolone/pyrazinedione group, but is absent in the three operons known to produce MB with an N-terminal oxazolone group (Semrau *et al.*, 2020 ▸), suggesting that MbnF is involved in the formation of the pyrazinedione/imidazolone group. To gain insight into the potential role of MbnF in MB biosynthesis, the crystal structure of MbnF was determined.

## 2. Materials and methods

### 2.1. Protein expression and purification

*Escherichia coli* codon-optimized *mbnF* from *Methylocystis* sp. strain SB2 was ordered from GenScript in a pET-41 vector (EMD Millipore) utilizing a C-terminal His_6_ tag (MbnF-His; Table 1 ▸). The cloning sites used were NdeI and XhoI. MbnF-His was expressed in *E. coli* BL21(DE3) cells. The cultures were grown at 37°C until the OD_600_ reached 0.5 and were then induced with 0.5 m*M* isopropyl β-d-1-thiogalactopyranoside and grown for an additional 16 h at 17°C. The cells were lysed with B-PER II (Thermo Fisher, Waltham, Massachusetts, USA) plus DNase and lysozyme. The cleared lysate was loaded onto a HisTrap column (MilliporeSigma, Burlington, Massachusetts, USA) equilibrated with buffer *A* [50 m*M* HEPES (VWR, Solon, Ohio, USA) pH 7.4, 50 m*M* NaCl]. The column was washed with three volumes of buffer *A* and eluted with a five-column-volume gradient from 0 to 500 m*M* imidazole in buffer *A* followed by three column volumes of buffer *A* with 500 m*M* imidazole. The eluate was collected in 2 ml fractions. The MbnF-containing fractions appeared yellow due to the presence of the associated flavin. The yellow fractions were dialyzed into 50 m*M* HEPES pH 7.4 and run on a Mono Q 10/100 GL anion-exchange column. The column was washed with three volumes of HEPES pH 7.4 and eluted with a five-column-volume gradient from 0 to 1 *M* NaCl followed by a three-column-volume 1 *M* NaCl wash with 50 m*M* HEPES pH 7.4 in all buffers. The yellow fractions were concentrated to 20 mg ml^−1^ using an Amicon Ultra-15 centrifugal filter (MilliporeSigma) and flash-frozen with 10% glycerol in liquid nitrogen. All columns were run on an ÄKTApure system.

### 2.2. Crystallization

Concentrated MbnF (20 mg ml^−1^) was subjected to high-throughput crystallization screening using a Mosquito LCP robot (SPT Labtech) with the commercially available screens JCSG-plus Eco, Ligand-Friendly Screen Eco, Morpheus and PACT premier Eco (Molecular Dimensions, USA) and the XP Screen (MiTeGen, USA). Crystals were observed in condition F2 of the PACT premier Eco screening kit (Table 2 ▸). The crystals from the PACT screen were cryoprotected with 15% glycerol and flash-cooled in liquid nitrogen.

### 2.3. Data collection and processing

The crystals were sent to the GM/CA 23-ID-B beamline at the Advanced Photon Source (APS) for screening and data collection. Native data sets were collected from crystals at a wavelength of 0.97931 Å; all data sets were indexed in space group *C*121. The data were integrated and scaled with *XDS* (Kabsch, 2010 ▸) within the *xia*2 (Winter, 2010 ▸) data-processing pipeline (Table 3 ▸).

### 2.4. Structure solution and refinement

MbnF phases were determined by molecular replacement (MR) using *Phaser* (McCoy, 2007 ▸). The successful search model was the aklavinone hydroxylase RdmE (PDB entry 3ihg, 39% sequence identity to MbnF; Lindqvist *et al.*, 2009 ▸), which was the closest homolog with known structure. *Autobuild* from the *Phenix* software suite (Liebschner *et al.*, 2019 ▸) was used to perform initial model building. *Coot* (Emsley *et al.*, 2010 ▸) was used for visualization and manual rebuilding. *Phenix.refine* was used for structure refinement (Table 3 ▸). The *super* algorithm in *PyMOL* (version 2.4.1; Schrödinger) was used for r.m.s.d. calculations. Coordinates and structure factors were deposited in the Worldwide Protein Data Bank as PDB entry 8fhj.

### 2.5. Molecular dynamics

Molecular-dynamics (MD) simulations were performed with *GROMACS* (Abraham *et al.*, 2015 ▸) using the AMBER99SB force field (Hornak *et al.*, 2006 ▸) and the SPC/E water model. The system was equilibrated with a 100 ps NVT simulation and a 100 ps NPT simulation followed by a 10 ns production MD run.

### 2.6. Enzyme assays

Assays were conducted in 500 µl reaction mixtures in 50 m*M* HEPES pH 7.4, 150 m*M* NaCl containing 100 nmol NADPH or 75 nmol NADH (EMD Millipore, Burlington, Massachusetts, USA) and 3.9 nmol (NADPH) or 2.5 nmol (NADH) MbnF, and reactions were carried out in a quartz cuvette (FireflySci, Northport, New York, USA). All reactions were monitored aerobically using a Cary 60 UV–visible absorption spectrophotometer (NADH, *A*_340_, ɛ = 6220 *M* cm^−1^). Reactions were carried out at 25°C and monitored for 50 min for the kinetics assay and overnight for the titration using the Cary *Kinetics* application. For NADPH oxidation assays, solutions were prepared anaerobically in a Coy anaerobic chamber (Coy Laboratory Products, Grass Lake, Michigan, USA) and allowed to degas overnight at 2°C. Reactions were initiated by the addition of NADPH followed by sparging with O_2_. For NADH oxidation assays, solutions were prepared aerobically with NADH and reactions were initiated by the addition of MbnF.

MbnA peptide (sequence IRIAKRITLNVIGRASARCASTCAATNG; ∼90% pure) was purchased from AnaSpec, Fremont, Calfornia, USA. MbnA experiments were performed using a mixture consisting of 1 mg ml^−1^ MbnA, 500 µ*M* MbnF, 250 m*M* NaCl, 50 m*M* HEPES pH 7.4. ESI-MS experiments were performed under acidic conditions on a SYNAPT G2-Si High-Definition Mass Spectrometer (Waters, Milford, Massachusetts, USA) with a Restek Ultra C4 5 µm 50 × 1 mm column and a water + 0.1% formic acid/acetonitrile + 0.1% formic acid gradient or under neutral pH conditions on an ACQUITY UPLC Protein BEH SEC column, 200 Å, 1.7 µm, 2.1 × 150 mm under isocratic conditions using 50 m*M* ammonium acetate buffer pH 6.6. The SYNAPT was run using standard parameters, scanning from 300 to 5000 Da. UV–Vis experiments were performed on a Cary 60 UV–visible spectrophotometer.

## 3. Results

### 3.1. Protein expression and purification

Codon-optimized (for *E. coli*) C-terminally His-tagged MbnF (MbnF-His) was expressed in *E. coli* BL21 (DE3) cells using a pET-41 vector and was purified by Ni–NTA and Mono Q chromatography. MbnF eluted from the HisTrap column with a small upstream shoulder and a long downstream tail (Supplementary Fig. S1). The presence of MbnF-His in the tail was apparent from the yellow color of the fractions and was supported by SDS–PAGE. MbnF-His eluted from the Mono Q column in a grouping of four peaks (Supplementary Fig. S1). This set of peaks ran as a single band on SDS–PAGE and as a single peak on gel filtration (Supplementary Fig. S1). The presence of MbnF-His as a grouping of peaks on the Mono Q column suggests that MbnF-His may form electrostatically distinct but related subspecies. Molecular weight and oligomeric state were assessed by gel-filtration chromatography, where MbnF-His eluted with an estimated molecular mass of 50 kDa compared with the computed mass of 59.17 kDa, suggesting that MbnF-His is a monomer in solution. The presence of FAD bound to MbnF-His throughout purification and crystallization was apparent by the yellow color of the solution and crystals; the buffers were not supplemented with FAD.

### 3.2. Crystallization

**Figure 2:**
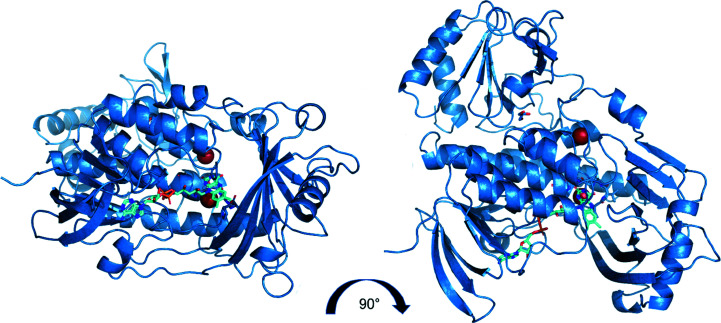
Two orthogonal views of the MbnF monomer structure. FAD is shown in cyan, with the two conserved bromide ions which co-crystallized with MbnF shown in red. A channel through the protein adjacent to the isoalloxazine ring of FAD is clearly visible in the left view.

Crystals of purified MbnF, which were greenish yellow in color, were obtained using the PACT premier Eco screen (Supplementary Fig. S2). Diffraction data were collected at the Advanced Photon Source (APS). The structure of MbnF was solved by molecular replacement (Fig. 2 ▸) and refined to a resolution of 2.61 Å (Table 3 ▸). The search model for molecular replacement was aklavinone hydroxylase (RdmE). RdmE is a flavin monooxygenase (FMO) that catalyzes the hydroxyl­ation of aklavinone to ɛ-rhodomycinone using NAPDH as a reductant (Lindqvist *et al.*, 2009 ▸). RdmE is 39% identical to MbnF over 96% of its length and superimposes with an r.m.s.d. of 3.2 Å (Supplementary Fig. S3). All three monomers of the asymmetric unit bound FAD in the same general conform­ation: the ‘in’ conformation where the FAD is less solvent-exposed and in a better position to interact with substrate compared with the ‘out’ conformation (Figs. 2 ▸ and 3 ▸; Ryan *et al.*, 2008 ▸). There are two MbnF structure predictions in the *AlphaFold* database (Jumper *et al.*, 2021 ▸; Varadi *et al.*, 2022 ▸), both from *Methylosinus* sp. sav-2, with 58% and 55% identity to the SB2 MbnF sequence used in this study. These two structures have r.m.s.d.s of 7.0 and 3.6 Å, respectively, relative to the structure reported here, but the overall topology is similar (Supplementary Fig. S4). However, the *AlphaFold* model does not include the FAD coenzyme.

### 3.3. Oligomeric state

MbnF-His eluted as a monomer from size-exclusion chromatography (Supplementary Fig. S1), suggesting that the monomeric form is physiologically relevant. However, when the crystal packing was examined it was found that chain *A* interacts with a twofold-related copy of chain *A* (Supplementary Fig. S5). This interface buries 2 × 1275 Å^2^. Surface areas greater than 2 × 856 Å^2^ have been found to predict physiologically relevant interfaces with a 15% error rate, according to a previous survey (Ponstingl *et al.*, 2000 ▸). Moreover, a nearly identical interface is observed between chain *B* and a symmetry-related copy of chain *C*: the *A*–*A* dimer and the *B*–*C* dimers superimpose with an r.m.s.d. of only 0.56 Å for 530 C^α^ pairs. The substantial binding area together with the presence of the dimer in two crystallographically non-equivalent arrangements suggest that the dimer might be important under *in vivo* conditions even if it is not directly observed by size-exclusion chromatography.

### 3.4. Structural features

Bromide ions were assigned to several electron-density features. The density was initially assigned to water molecules, but after refinement residual positive density remained, suggesting that more electrons were needed to satisfy the map. These waters were replaced with bromide ions, consistent with the presence of 200 m*M* sodium bromide in the crystallization conditions. After refinement with the modeled bromide ions the residual density diminished.

**Figure 3:**
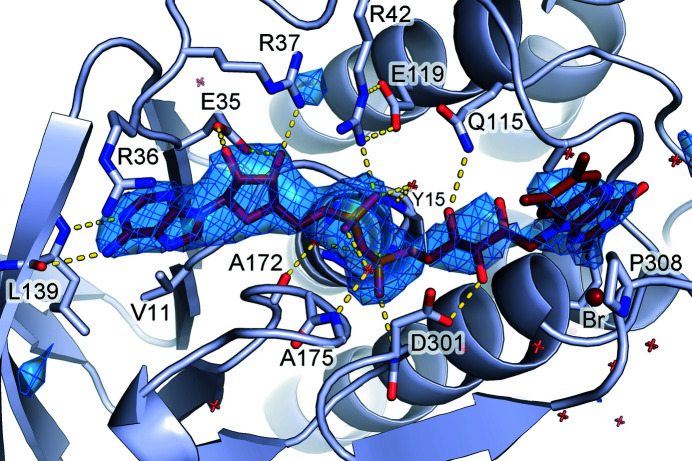
Close-up of the FAD-binding pocket of MbnF. Fit of FAD to a simulated-annealing omit map that was calculated with FAD atoms omitted. The difference map is displayed at 3.1σ. The residues that contact FAD are labeled. The yellow dashed lines indicate hydrogen bonds. The small red crosses represent water molecules.

**Figure 4:**
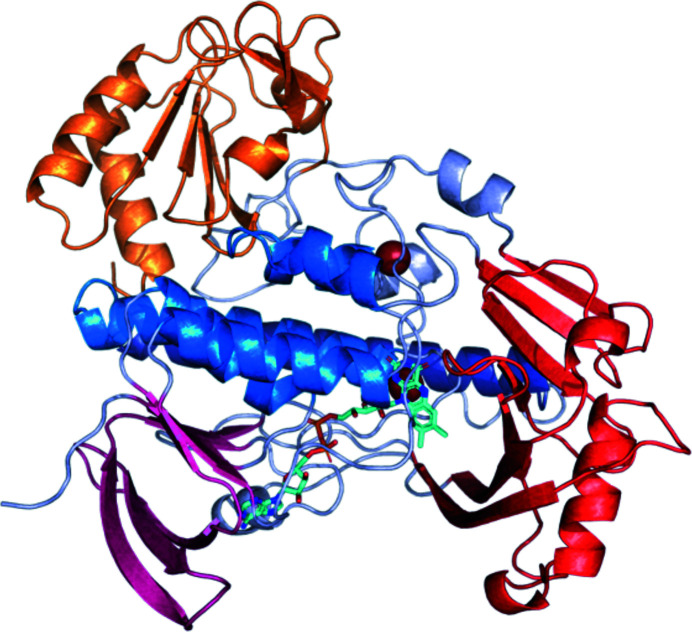
Structure of the MbnF monomer color-coded by structural domain with FAD shown in cyan: five-helix bundle; blue; β-sandwich coordinating the adenine moiety of FAD, pink; β-hotdog forming the putative active site, red; C-terminal domain featuring β–α–β and β–β–α motifs, orange; 30-amino-acid helix containing a central proline, purple.

With regard to the backbone, MbnF has four major structural motifs (Fig. 4 ▸ and Supplementary Fig. S6): a five-helix bundle that makes up the central core of MbnF and coordinates the isoalloxazine moiety of FAD, an N-terminal β-sandwich that coordinates the adenine moiety of FAD, a β-hotdog motif that forms a channel adjacent to the flavin ring, and finally a domain featuring β–α–β and β–β–α motifs connected to the 30-amino-acid helix by an ∼50-residue loop region. FAD is not covalently bonded to MbnF but rather is associated by numerous noncovalent interactions (Fig. 3 ▸). Additionally, adjacent to the helix bundle is a 30-amino-acid helix at the protein surface. Interestingly, this helix has a proline at position 13, a residue that occurs relatively infrequently in helices of soluble proteins (Cordes *et al.*, 2002 ▸). The presence of this residue introduces a kink of ∼20°, which is fairly typical of proline-induced helical kinks (Barlow & Thornton, 1988 ▸). This proline is not present in the RdmE search model. The two phosphate moieties of FAD are positioned at the N-terminal end of helix 2, implying that the dipole of the helix may contribute to the coordination of FAD. In all three monomers of the asymmetric unit, electron-density features interpreted as bromide ions are observed coordinated between the isoalloxazine ring of the FAD and the N-terminal end of helix 2. The presence of these anions may indicate the coordination of negatively charged groups present in the substrate. It is also of note that adjacent to the isoalloxazine ring is a channel that appears to be large enough to accommodate a peptide. This is of interest as MbnA is a potential substrate of MbnF.

### 3.5. Molecular dynamics

**Figure 5:**
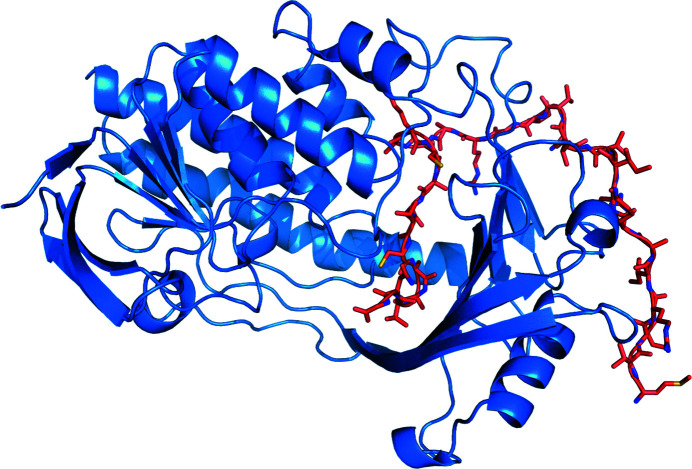
Molecular-dynamics simulation of the MbnF crystal structure with MbnA (red) docked in the active-site channel. This simulation shows that the channel is large enough to accommodate the MB peptide without clashes. However, further analysis of the contacts between MnbF and MbnA would be unreliable due to the short length of the simulation. The MD simulation was run in *GROMACS* using the AMBER99SB force field and the SPC/E water model. The structure was equilibrated with 100 ps NVT and NPT calculations followed by a 10 ns production run. The average r.m.s.d. was relatively flat by 4 ns.

**Figure 6:**
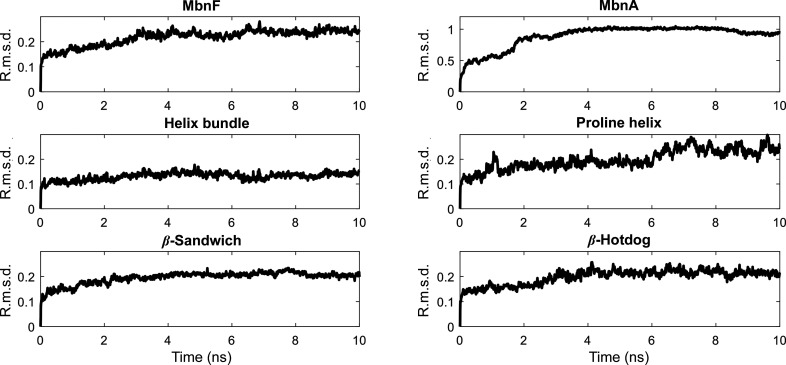
Backbone r.m.s.d. of MbnF–MbnA during the MD simulation. All graphs are plotted as r.m.s.d. (nm) against simulation time (ns) and all graphs are scaled from 0 to 0.3 nm, except for that for MbnA, which is scaled from 0 to 1.2 nm. From top to bottom and from left to right are the r.m.s.d. for full MbnF, MbnA (the region not docked into the MbnF channel underwent large conformational changes), the helix-bundle domain, the β-sandwich motif adjacent to the adenine moiety of FAD and the β-hotdog motif adjacent to the alloxazine ring of FAD.

To test whether this channel could accommodate a peptide, MbnA was manually docked into the active-site channel of MbnF residues 1–370 (this removes the C-terminal domain and the loop region following helix 22, with FAD removed as well), with the C-terminal end of MbnA in the region adjacent to the position where the isoalloxazine ring of FAD would normally be. This MbnF–MbnA complex was run for 10 ns in an MD simulation to test for any steric clashes and for the plausibility of this channel accommodating a peptide (Fig. 5 ▸). While there are no obvious interactions between MbnA and MbnF, 10 ns is likely to be too short a simulation for these to develop (Bowman, 2016 ▸). It is interesting to note, however, that while most of the MbnF domains reached a relatively stable r.m.s.d. after 2–4 ns (Fig. 5 ▸), the 30-residue helix containing a proline did not stabilize after 10 ns (Fig. 6 ▸ and Supplementary Fig. S7). It is possible that this helix was stabilized by the crystal environment as there is clear electron density for this helix, but it is not clear what other structure it may adopt in solution. It is of note that this helix is directly connected to a ∼50-residue loop region.

### 3.6. Functional characterization

**Figure 7:**
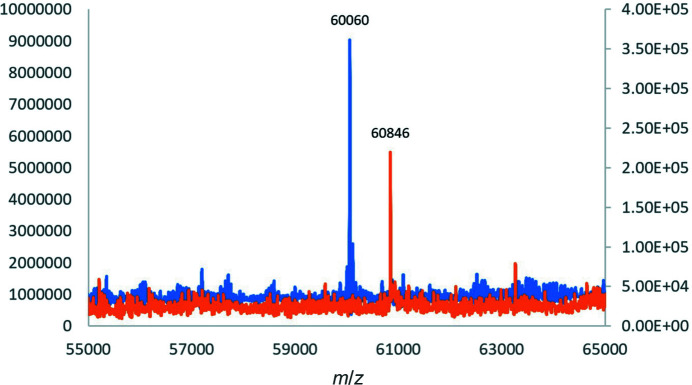
MS spectra of MbnA + MbnF on a C4 column (blue) and SEC column (orange). Under acidic conditions MbnA separated from MbnF on the C4 column and only MbnF is observed in this molecular-mass range. Under the neutral pH conditions of the SEC column MbnA remained bound to MbnF.

Putatively thought to be a flavin-dependent monooxygenase, the catalytic details of MbnF are unknown. In the presence of MbnF, NADH and NADPH were oxidized at rates of 0.1 and 0.7 min^−1^, respectively (Supplementary Fig. S8). This preference for NADPH over NADH has been observed in other FMOs (Paul *et al.*, 2021 ▸). MbnA also appears to bind MbnF (Fig. 7 ▸). Upon the incubation of MbnF with MbnA, a shift of 786 Da was observed in the ESI-MS corresponding to the mass of the core sequence in MB-SB2, *i.e.* RCASTCAA (Krentz *et al.*, 2010 ▸). This result suggests that MbnA binds to MbnF and tentatively suggests that MbnF might be used for hydrolysis of the leader sequence as well as the C-terminal amino acids from the core peptide in MbnA. Alternatively, however, fragmentation during ionization could account for the cleavage of MbnA. We also considered that MbnF might be involved in one of the post-translational modifications of the core MbnA peptide. UV–Vis spectroscopy (which can detect the imidazolone and oxazolone rings of MBs) provided no evidence for changes in MbnA following the incubation of MbnA with MbnF and NAD(P)H. However, if MbnF does modify MbnA, the reaction might take place later in the biosynthetic pathway of MB following other post-translational modification(s).

## 4. Discussion

Here, the X-ray crystal structure of MbnF from *Methylocystis* sp. strain SB2 is presented at a resolution of 2.6 Å, which was solved by molecular replacement using the aklavinone hydroxylase RdmE (PDB entry 3ihg, 39% identity) as a search model. MbnF forms a homodimer in two independent observations, but was observed to be monomeric by SEC. MbnF contains multiple structural features consistent with a type A FMO, including multiple FAD-binding motifs. Each monomer in the asymmetric unit crystallized with 2–6 bromide ions in eight unique sites, two of which are conserved. It is interesting to note that there is a large channel through MbnF with the isoalloxazine ring of the FAD at one end and one of the two conserved bromide ions at the other end. The other conserved ion is directly adjacent to the isoalloxazine ring of the FAD (3.3 Å from N1). The presence of these ions indicates the possibility of negative charges being utilized in the coordination of the substrate.

MbnF is present in all *mbn* operons known or proposed to encode an N-terminal imidazolone/pyrazinedione group and may be responsible for modifying an N-terminal intermediate oxazolone group to a pyrazinedione/imidazolone group, possibly via the addition of a hydroxyl group, which is the most common activity of type A FMO enzymes (Paul *et al.*, 2021 ▸). It is possible that the partial charge of the ketone in the oxazolone is used to coordinate the substrate in the active site, although this remains speculative. Further, manual docking of MbnA into the channel adjacent to the isoalloxazine of the FAD followed by a 10 ns MD simulation shows that this proposed active-site channel is large enough to accommodate this peptide. More extensive simulations need to be performed that include the FAD to assess any coordinated interactions between the peptide and MbnF. The exact reaction that is catalyzed by MbnF still remains unclear.

The results presented here tentatively suggest that MbnF may somehow be involved in the hydrolysis of both the leader peptide as well as the C-terminal amino acid(s). One to three C-terminal amino acid(s) of the precursor polypeptide are not observed in the final MB from *mbn* operons containing *mbnF* (DiSpirito *et al.*, 2016 ▸; El Ghazouani *et al.*, 2012 ▸; Krentz *et al.*, 2010 ▸). Here, MS data suggested the loss of both the leader peptide as well as the three C-terminal amino acids following incubation of MbnA and MbnF, although this might have been due to fragmentation during MS rather than hydrolysis. The last two amino acids in MbnA from *Methylocystis* sp. strain SB2 have never been observed in purified MB-S2 samples and the third amino acid from the C-terminal end is only observed in a fraction of the final product (DiSpirito *et al.*, 2016 ▸; Krentz *et al.*, 2010 ▸). In structurally characterized MBs from *mbn* operons not containing *mbnF*, only one amino acid is occasionally missing from a fraction of the final product (Bandow *et al.*, 2011 ▸; El Ghazouani *et al.*, 2011 ▸). Thus, although homologs of MbnF are not known to have peptidase activity, the MbnF reaction might be needed for trimming of the MbnA precursor peptide to occur.

Here, we present a structure of MbnF from *Methylocystis* sp. strain SB2 and evidence supporting the plausibility of MbnF playing a catalytic role in MB maturation. MbnF is able to oxidize NAD(P)H consistent with its role as an FMO, and the putative active-site channel of MbnF is able to accommodate a peptide such as MbnA. Further investigation is required to substantiate the role of MbnF in the maturation process of MB of *Methylocystis* sp. strain SB2 and its catalytic mechanism.

## Supplementary Material

PDB reference: MbnF, 8fhj

Supplementary Figures. DOI: 10.1107/S2053230X23003035/no5198sup1.pdf

## Figures and Tables

**Table 1:** Cloning details

DNA source	Synthetic codon-optimized DNA
Cloning vector	pET-41a
Expression vector	pET-41a
Expression host	*E. coli* BL21 DE3
Complete DNA sequence of the expression product	ATGAGCGTGGAGCGTGTGCCGGTTCTGATCGTTGGTGCGGGTTATGCGGGTCTGAGCGCGGCGACCCTGCTGGCGTGGCGTGGTGTGCCGTGCCGTCTGGTTGAACGTCGTGCGAGCACCAGCCGTCTGCCGAAGGCGCACGGTATTAACCGTCGTAGCATGGAAGTGCTGCGTGTGGTTCCGGGTCTGGAAGATGCGCTGTTTGCGGCGAGCCGTGCGGGTGCGAACGAGAGCACCCTGATCATTGCGGAAAGCGTGACCAGCCCGCCGATTGAGACCCTGGTTACCAAAATTAGCCTGGATGCGACCCCGGTTAGCCCGAGCCGTATCTGCACCGCGGGTCAGGATCGTGTGGAGCCGGTTCTGCTGCGTTTTGCGCGTGAAAACGGTGCGGATGTTCGTTTCAGCACCACCCTGGAACGTTTTAGCCAGCGTGACGATGGCGTGGACGCGATCCTGCGTGATGAGGCGAGCGGTCAAGAAACCACCGTTCTGGCGGACTACATGATTGCGGCGGATGGTGCGGGTGGCACCATTCGTGACGTGGGTGGCGTTAAGATGGAGGGCCCGGGTGTGCTGGCGGATACCATCAGCGTTCTGTTCGAAGCGGACCTGGATAGCATTCTGCCGGGTGGCGGTTTTGCGCTGTACTATCTGCGTAACCCGGCGTTCAGCGGCGCGTTTGTGACCTGCGACGAGCCGAACCACGGTCAGATCAACATTGAATATGACAGCACCCGTGATCAAGCGAGCGACTTCGATGAGGAACGTTGCGAAGCGCTGGTTCGTCAAAGCCTGGGCGTGGCGGACCTGGCGGTTAAAATCCTGGATATTCGTCCGTGGCAAATGGCGGCGCTGCTGGCGGACCGTATGAGCTTCGGTCGTGTGTTTCTGGCGGGTGATTGCGCGCACATCACCCCGCCGGTTGGCGGTCTGGGCGGTCAGACCGCGATTCAAGATGCGGCGGATCTGGCGTGGAAGCTGGCGCTGGTTGTGAAAGGTCAAGCGGCGCCGACCCTGCTGGACAGCTACGAGATCGAACGTCGTCCGGTGGCGCGTATCGCGATTGCGCGTAGCATTGCGAACTACGTTGAGCGTCTGCTGCCGGACCGTCAAGATATCCGTATTCGTGAGGACGAATATGGTCTGCTGGAAACCGCGATGGGCTACCGTTATCGTAGCGACGCGATCATTGCGGATGAGTTCGACGATGGTGCGTGCGTGGAAGATCCGCTGCGTCCGAGCGGTGCGCCGGGTACCCGTCTGGCGCACGTTTGGCTGCGTCGTGGTGAGGAAACCATCAGCAGCCACGACCTGATTGGTCGTGATTTCATGCTGTTTACCGGTCCGGATGGCGGTGATTGGATTGAGGCGGCGCGTCGTATTGCGCTGCGTAGCAAAGCGCCGCTGGGTGTGTGCCGTCTGGGCTTCGACGTTGACGATCCGGAGGGTCTGTTTCTGCCGCGTCTGCGTATCAGCCCGGAAGGTGCGCTGCTGGTGCGTCCGGACGGCTATATTGCGTGGCGTAGCCGTGGTCGTAGCCCGGATCCGTTCGCGACCCTGGAAGCGAGCTTCGCGCGTGTTCGTGGCTTTGACACCGGTCAAAGCAGCGGTAGCCATGCGGCGGCGTTTGCGGATGCGAGCGGTAGCGGTCATCACCACCACCACCACTAA

**Table 2:** Crystallization

Method	Vapor diffusion, sitting drop
Plate type	96-3 low-volume reservoir INTELLI-PLATE (Art Robbins Instruments)
Temperature (K)	291
Protein concentration (mg ml^−1^)	20
Buffer composition of protein solution	50 m*M* HEPES pH 7.4, 50 m*M* NaCl
Composition of reservoir solution	100 m*M* bis-Tris propane pH 6.5, 200 m*M* sodium bromide, 20%(*w*/*v*) PEG 3350
Volume and ratio of drop	105 nl:105 nl
Volume of reservoir (µl)	50

**Table 3:** Data-collection and processing statistics Values in parentheses are for the highest resolution shell.

Space group	*C*121
*a*, *b*, *c* (Å)	148.35, 96.77, 135.21
α, β, γ (°)	90.00, 116.41, 90.00
Resolution (Å)	59.95–2.61
Data completeness (%)	99.6
〈*I*/σ(*I*)〉	1.19 [at 2.61 Å]
*R*, *R*_free_	0.204, 0.254
*R*_free_ test set	1999 reflections [3.82%]
Wilson *B* factor (Å^2^)	66.7
Bulk solvent *k*_sol_ (e Å^−3^), *B*_sol_ (Å^2^)	0.40, 63.7
*L*-test for twinning	〈|*L*|〉 = 0.51, 〈*L* ^2^〉 = 0.34
Estimated twinning fraction	No twinning to report
*F*_o_, *F*_c_ correlation	0.96
Total No. of atoms	12534
Average *B*, all atoms (Å^2^)	71.0
CC_1/2_	0.996 (0.42)
*R*_merge_	0.1243 (1.679)
*R*_p.i.m._	0.0780 (1.02)
