# Supplementary material for: Crystal structure of MbnF: an NADPH-dependent flavin monooxygenase from Methylocystis strain SB2
Source: Acta Crystallogr F Struct Biol Commun. 2023 May 5;79(Pt 5):111–8. doi: 10.1107/S2053230X23003035 (PMC10167746; doi:10.1107/S2053230X23003035)
Supplement: Supplementary file 1 [file f-79-00111-sup1.pdf]

STRUCTURAL BIOLOGY COMMUNICATIONS

Volume 79 (2023)

Supporting information for article:

Crystal structure of MbnF: an NADPH-dependent flavin monooxygenase from *Methylocystis* strain SB2

Andrew Stewart, Philip Dershwitz, Charles Stewart, Michael R. Sawaya, Todd O. Yeates, Jeremy D. Semrau, Hans Zischka, Alan A. DiSpirito and Thomas A. Bobik

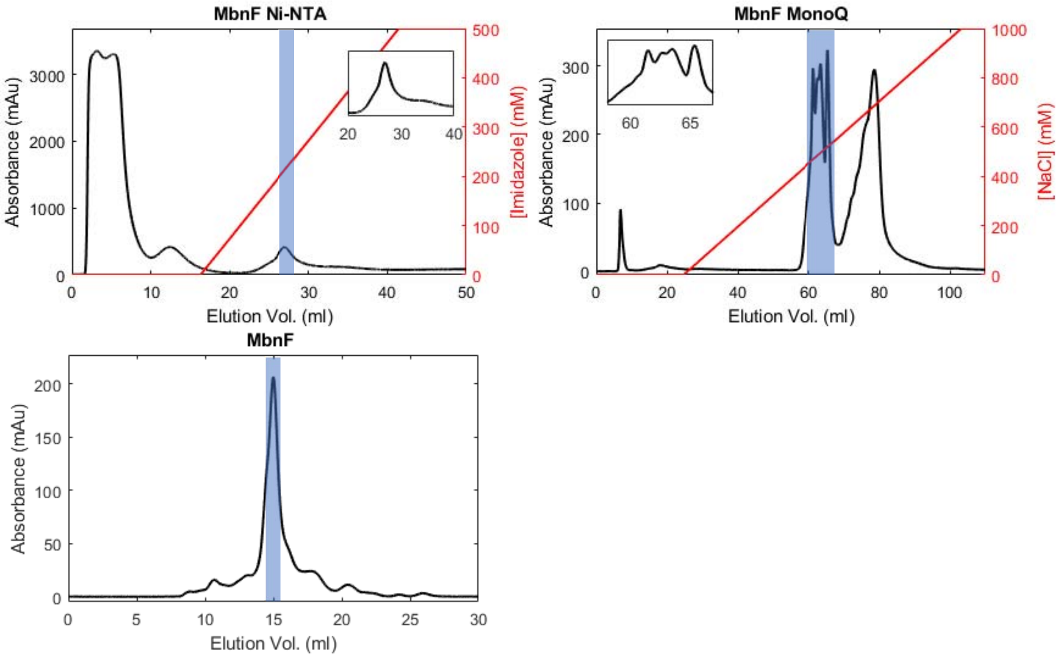

Figure S1 Chromatograms from the MbnF purification. MbnF fractions are highlighted in blue. All columns were run on an ÄKTA Pure system. (Top left) Elution profile of MbnF cleared lysate on HisTrap column. HisTrap column was equilibrated and run with 50 mM Hepes pH 7.4 and 50 mM NaCl, using an imidazole gradient from 0 – 500 mM. MbnF eluted at 27 ml (~200 mM imidazole). Inset shows MbnF peak. Absorbance shown in black, and buffer B concentration shown in red. (Top right) Elution profile of MbnF obtained after the HisTrap column on a MonoQ 10/100 GL column. The column was equilibrated and run with 50 mM Hepes pH 7.4, using an NaCl gradient from 0 – 1 M. MbnF eluted at 60 ml (~450 mM NaCl). Inset shows MbnF peak. Absorbance shown in black, and buffer B concentration shown in red. (Bottom left) Chromatogram of MbnF on Superdex 200 10/300 GL. The column was equilibrated and run with 50 mM Hepes pH 7.4, 150 mM NaCl. MbnF eluted at 14.3 mL as the primary peak.

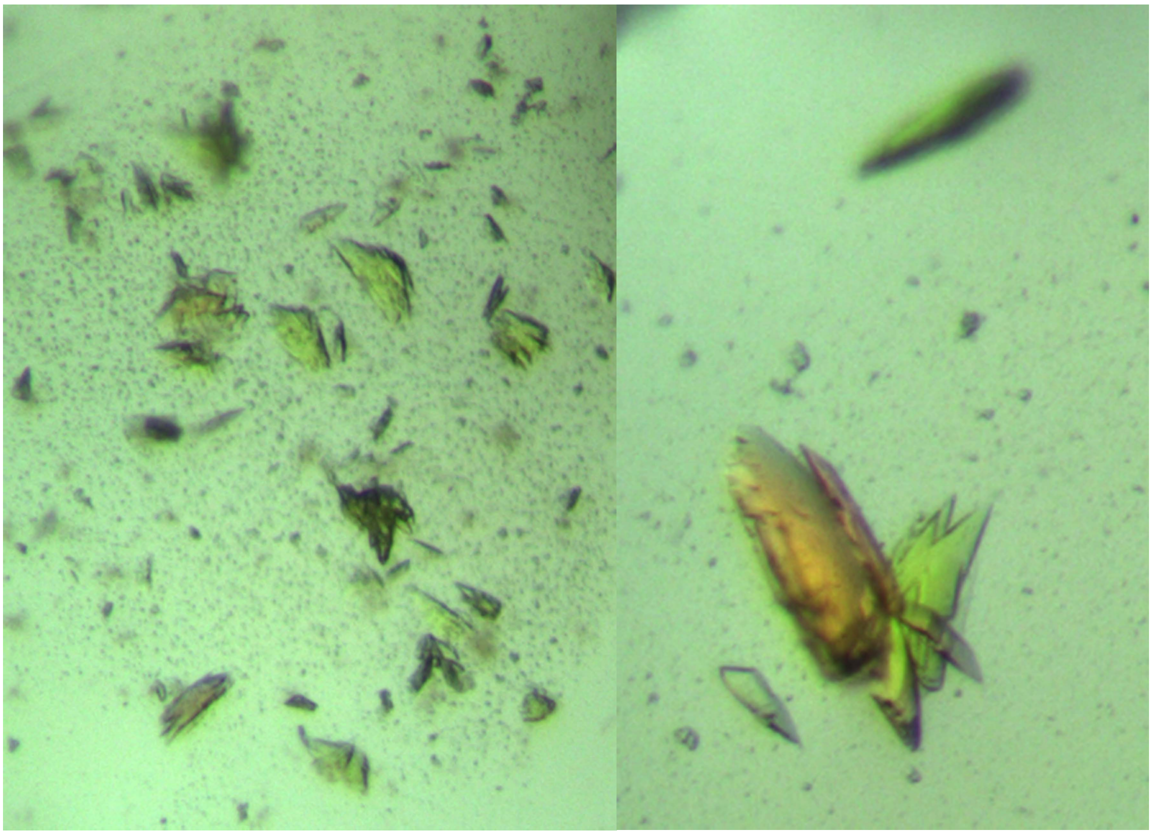

Figure S2 MbnF Crystals used for X-ray diffraction. Crystals were grown in 20% PEG 3350, 0.1 M Bis-Tris propane pH 6.5, 0.2 M sodium bromide (PACT premier Eco crystal screen well F2), and grown at 18°C.

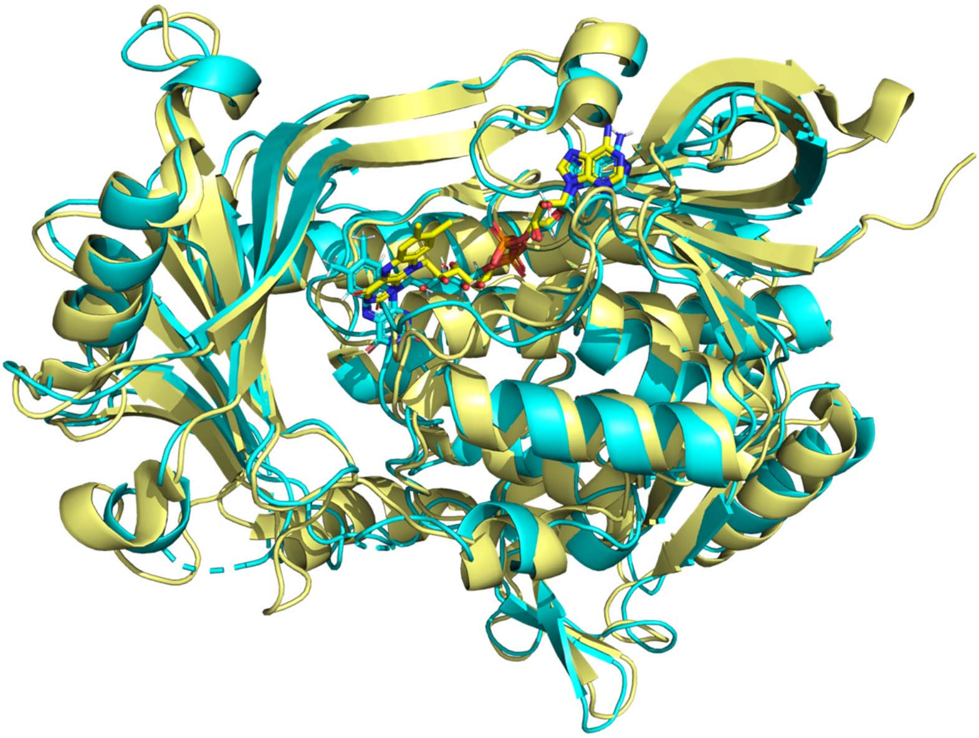

Figure S3 . MbnF superimposed on RdmE, its closest structural homolog. The overlay shown is chain A of RdmE aligned with chain A of MbnF using Pymol software. The RMSD is 3.2 Å.

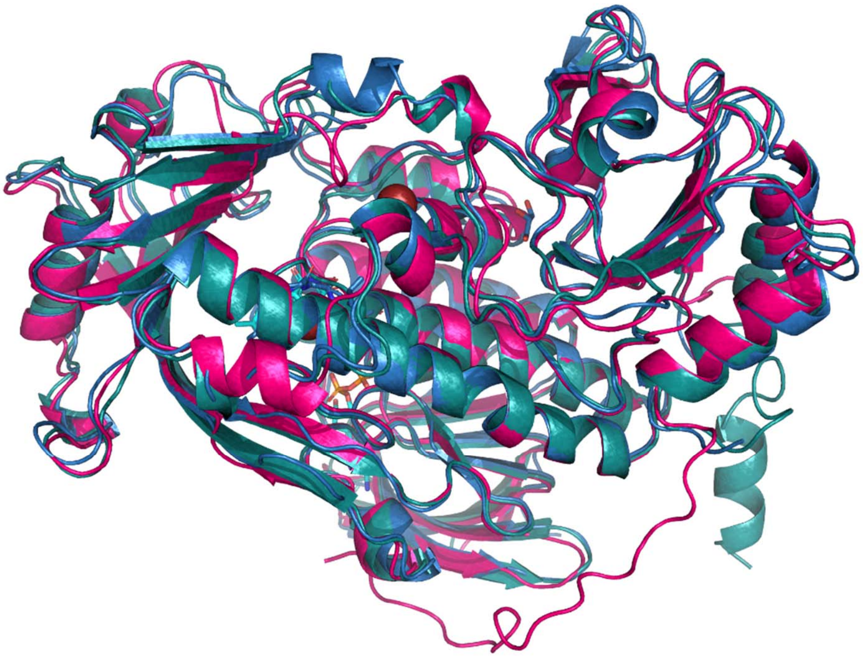

Figure S4 Crystal structure of MbnF (blue) aligned with the AlphaFold predictions for two MbnF proteins from *Methylosinus* sp. sav-2. The two proteins are 58 and 55% identical to the MbnF sequence from *Methylocystis* sp. Strain SB2 used in this study, and have relative RMSDs of 7.0 and 3.6 Å respectively.

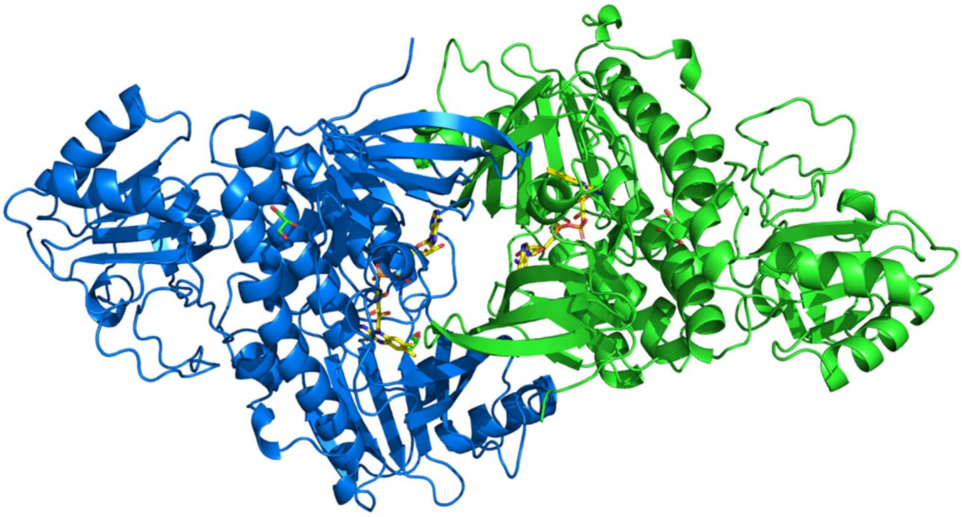

Figure S5 Crystallographic dimer of MbnF. Similar interactions are seen between the symmetry related A-A dimer and the B-C dimers. The A-A dimer is shown.

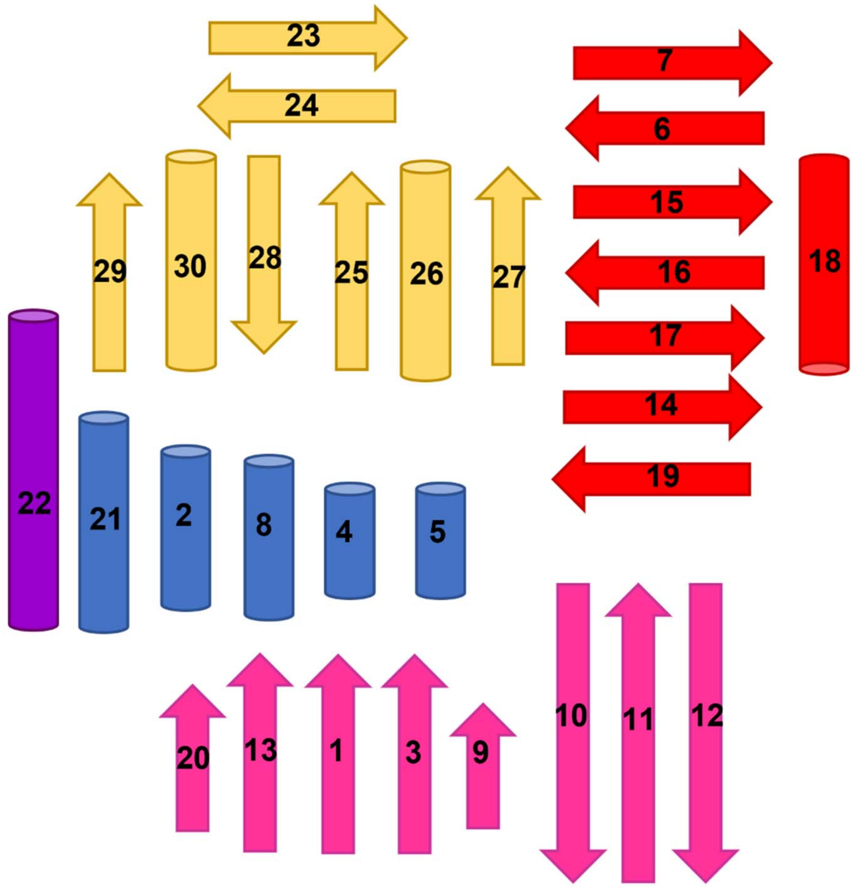

Figure S6 Topology of MbnF structural domains color-coded by domain. Numbering from 1 to 30 shows the relative position in the primary sequence from N to C terminals. It is of note that only the yellow domain is fully isolated from the other domains with ~50 residues between element 22 and 23. The other three domains all have inter-connected elements tying these domains together.

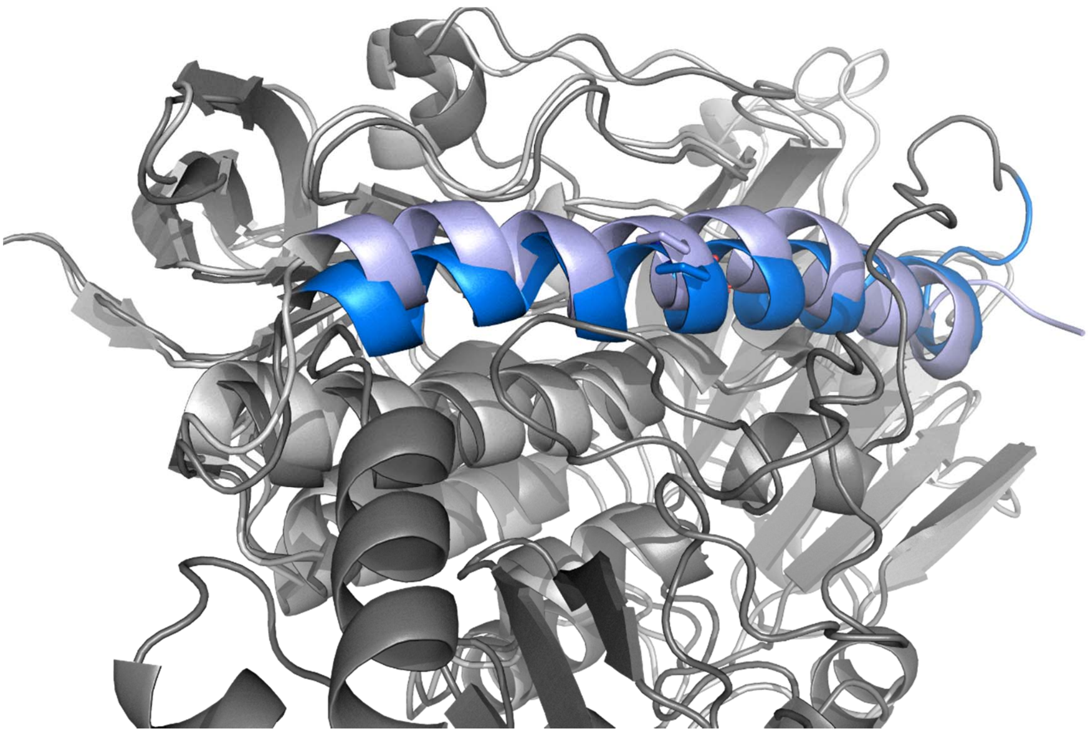

Figure S7 During MD simulation helix 22 of MbnF did not stabilize after 10ns. Close up of helix 22 of MbnF which contains a central proline (shown as sticks for clarity). The helix from the crystal structure is shown blue, and the helix after a 10 ns MD simulation is shown in silver. While there is an apparent deviation from the crystal structure, it is of note that even after the 10 ns simulation, this helix had not yet come to equilibrium. A longer simulation would be required to assess the equilibrium state of this helix. It should be noted that the C-terminal domain and ~50 residue loop region connected to helix 22 was removed for the MD simulation.

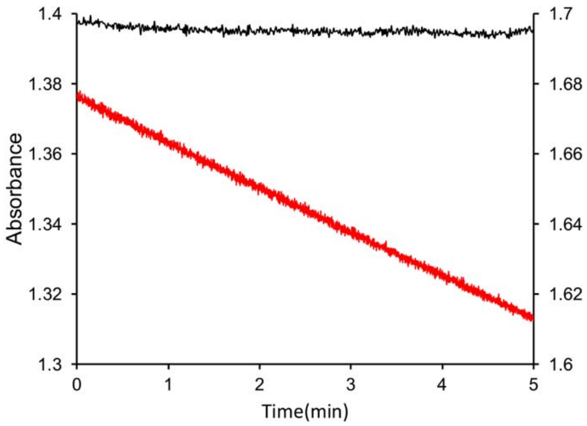

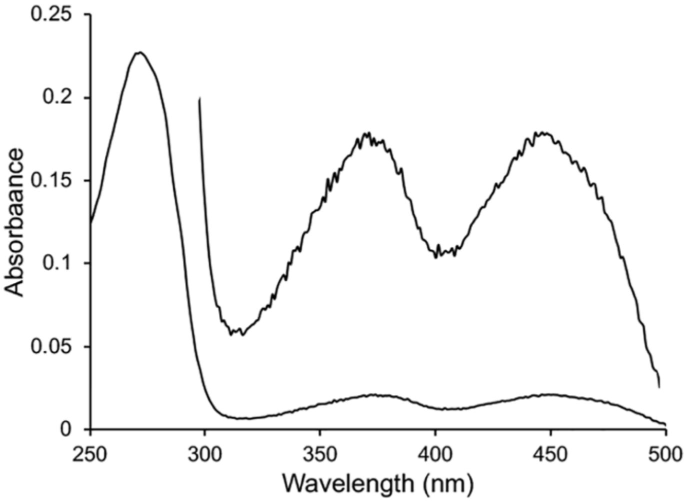

Figure S8 (Top) Kinetics assay showing the change in A_{340} for NADPH in the presence (red) and absence (black) of MbnF. (Bottom) UV-visible spectra of MbnF, with the flavin region zoomed in (inner trace).
